# Editorial: Underlying mechanisms transitioning seeds to seedlings

**DOI:** 10.3389/fpls.2024.1503165

**Published:** 2024-10-30

**Authors:** Muhammad Awais Farooq, Ewa Marzena Kalemba, Alma Balestrazzi

**Affiliations:** ^1^ Department of Agricultural and Food Sciences (DISTAL), Alma Mater Studiorum, University of Bologna, Bologna, Italy; ^2^ Institute of Dendrology, Polish Academy of Sciences, Kórnik, Poland; ^3^ Department of Biology and Biotechnology ‘L. Spallanzani’, University of Pavia, Pavia, Italy

**Keywords:** seed germination, seedling development, seed priming, omics in seed development, seed dormancy

Successful seed germination marks a critical phase in the life cycle of a plant where the dormant seed transitions into a seedling capable of independent growth. The ability of the seed to transform into a seedling and eventually develop into a mature plant determines the reproductive and ecological success of the plant in the environment and, from a human perspective, economic profit. This transition is a highly coordinated process governed by an intricate interplay of omics networks that ensure the proper utilization of stored resources and the activation of developmental programs. Omics networks involving phytohormones, proteins, metabolites, specific transcription factors, epigenetic remodeling of chromatin, and small RNAs constitute the molecular control of the seed germination process which is modulated by the mechanical forces between seed tissues and external factors such as light, water, and oxygen (Carrera-Castaño et al., 2020; Baud et al., 2023). A complete understanding of the mechanisms underlying the transition from seeds to seedlings is crucial to managing the genetic resources via *in situ* and *ex situ* conservation programs and for sustainable agriculture because seedling emergence and establishment are considered to be the most vulnerable stages of plant growth and development in global warming scenarios (Badano and Sánchez-Montes de Oca, 2022). In this Research Topic “*Underlying mechanisms transitioning seeds to seedlings*” eight articles have been published that cover the main themes of the Research Topic: gene regulatory network, physiological processes regulating seed germination under stress conditions, and interaction between the internal and external environment driving the seed germination.

Selection of specific genotypes and sustainable seed priming techniques in response to water shortage stress leads to the cultivation of efficient, environmentally friendly crops. Dueñas et al. demonstrated that poly-gamma-glutamic acid (γ-PGA), denatured γ-PGA (dPGA) and iron pulses are able to enhance the resilience to drought stress in four Italian rice varieties and indicated one rice variety as highly stress sensitive. The enhancement was manifested by altered expression of genes involved in DNA damage response, antioxidant defense mechanism, drought stress response, amino acid transport, and iron homeostasis. In another study, a novel seed treatment using 2-(N-methyl benzyl aminoethyl)-3-methyl butanoate (BMVE) was reported by Dharni et al. to consistently improve seed development subsequent seed germination, seedling vigor, and stress tolerance in the case of rice and wheat. Transcriptomic analysis revealed that changes in reactive oxygen species (ROS) scavenging, hormone signaling and stress response pathways are the molecular mechanisms determining enhanced seedling growth and productivity of rice and wheat. Both articles suggested that priming methods could be a practical seed treatment to mitigate the impact of climate change on enhancing crop growth. In the third study related to seed priming, the effect of melatonin (MT), pollen polysaccharide (SF), and 14-hydroxyed brassinosteroid (14-HBR) on the growth of kiwifruit seedlings was investigated by Zhang et al. MT, SF, and 14-HBR were found to increase leaf chlorophyll content, photosynthetic capacity, and antioxidant enzyme activities, to enhance dry shoot biomass and modify the soil parameters, as reflected in the activities of soil enzymes, the abundance of bacteria and the content of available K and organic matter. In conclusion, 14-HBR combined with MT was the most efficient in promoting rhizosphere bacterial distribution, nutrient absorption and plant growth.

Transcriptome study approaches enable the complex mapping of a genome to various phenotypes, developmental stages, and environmental factors. Since literature data present little information regarding seedling establishment in heteromorphic species, the work of Arshad et al. on the dimorphic diaspore model *Aethionema arabicum* is particularly valuable. Transcriptomics of the natural dimorphic diaspores, the mucilaginous seeds and the dispersed indehiscent fruits during late germination revealed morph-specific differential expression of hormone regulators, water and ion transporters, nitrate transporters and assimilation enzymes, and cell wall remodeling protein genes. In another article, Slocum et al. analyzed the expression of genes encoding enzymes and transporters of nucleotide metabolism in pyrimidine-starved Arabidopsis seedlings after uridine supplementation. Genome-wide transcriptional profiling revealed that stress responses, cellular responses to hypoxia or pathogens, auxin, and calcium (Ca^2+^) homeostasis and signaling, root development, cell wall loosening during growth, plastid development, and photosynthesis are the main processes impacted by changes in pyrimidine availability. Additionally, transcription factors involved in the abiotic and biotic stress responses appeared to be important agents controlling key steps in pyrimidine and purine synthesis and salvage. Although the mechanisms of ABA-mediated seed germination inhibition have been previously investigated, Xiang et al. demonstrated that the gene encoding pectin methylesterase 31 (*PME31*) is transcriptionally inhibited by ABI5, the transcription factor involved in abscisic acid (ABA) signaling, and therefore negatively regulates ABA-mediated seed germination inhibition. The authors suggested that tissue-specific pectin methylesterification may play a role in the regulation of seed germination because *PME31* was expressed in the embryo, leaf vasculature, and primary root of Arabidopsis.

Studies of mutants of *Arabidopsis thaliana* are providing crucial findings in seed science. The role of arogenate dehydratase (ADT), which is involved in phenylalanine synthesis, in the switch from heterotrophic growth to light-driven autotrophy is addressed in an article by Muhammad et al. who investigated the impact of light conditions and specific hormone responses in *A. thaliana* seedlings using six *adt* mutants. The authors demonstrated that phenylalanine pools are specific to tissues with respect to age and level of development and are implicated in the regulation of seed germination, hypocotyl elongation, root development, and plastid development.

Huang et al. investigated the hydroponic-based cultivation of two lettuce varieties. The authors demonstrated that pH, nutrient concentration (Hoagland solution), light intensity, cultivation substrate hardness, and porosity affect the lettuce germination time and success rate. Their research contributed to a better understanding of the hydrogel-aided lettuce germination and may help to optimize its use in agricultural production.

The collection of eight articles included in this Research Topic demonstrates the complexity of the seed-to-seedling transition and highlights the significance of investigating the mechanisms that regulate this process. These studies have contributed to a better understanding of the factors that determine seedling emergence and performance, which can facilitate successful and sustainable agriculture, horticulture and forest practices. This Research Topic delivers a key message: expanding current seed treatments to include novel approaches and tools is a sustainable strategy to reinforce crop resilience to climate change whereas exhaustive omics profiles can disclose the molecular basis of specific seed features, useful to improve germination performance in response to stress.
